# Piezochromic Nanomaterials: Fundamental Mechanisms, Advances, Applications, and Future Prospects in Solar Cell Engineering

**DOI:** 10.3390/nano16030175

**Published:** 2026-01-28

**Authors:** Xingqi Wu, Haoyuan Chen, Yang Luo, Jiang Yu, Yongan Wang, Kwang Leong Choy, Zhaodong Li

**Affiliations:** 1 Extra High Voltage Company, State Grid Hubei Electric Power Co., Ltd., Wuhan 430050, China; 2 Suzhou Key Laboratory of Advanced Sustainable Materials and Technologies, The Environmental Research Center, Duke Kunshan University, Kunshan 215316, China; 3 School of Integrated Circuits, Wuhan University, Wuhan 430000, China; 4 The Institute of Technological Sciences, Wuhan University, Wuhan 430000, China; 5 School of Power and Mechanical Engineering, Wuhan University, Wuhan 430000, China

**Keywords:** piezochromic nanomaterials, strain-programmable, photovoltaic technologies, mechanical–optoelectronic coupling

## Abstract

Piezochromic nanomaterials, whose optical responses can be reversibly tuned by mechanical stimuli, have recently gained prominence as versatile platforms for strain-programmable light–matter interactions. Their mechanically responsive band structures, excitonic states, and defect energetics have enabled a wide range of optoelectronic demonstrations—including pressure-tunable emitters, reconfigurable photonic structures, and adaptive modulators—which collectively highlight the unique advantages of mechanical degrees of freedom for controlling optical functionality. These advances naturally suggest new opportunities in photovoltaic technologies, where experimentally validated phase stabilization and defect reorganization under low-strain thin-film conditions could address long-standing limitations in solar absorbers and device stability. Meanwhile, stress-mediated bandgap tuning—largely inferred from high-pressure laboratory studies—presents a conceptual blueprint for future adaptive spectral response and structural self-monitoring. However, the application of these mechanisms faces a major challenge in bridging the magnitude gap between GPa-level high-pressure phenomena and the low-strain regimes of realistic operational environments. Future development requires advances in low-threshold responsive materials, innovative strain-amplifying device architectures, and the pursuit of intelligent, multi-functional system integration.

## 1. Introduction

Piezochromism—the reversible color change induced by mechanical stimuli such as pressure or stress—has garnered considerable attention in recent years due to its transformative potential across diverse applications, including pressure sensing, smart packaging, flexible electronics, and adaptive optoelectronics [1,2,3]. This optical phenomenon, arising from the mechanical deformation of materials, enables real-time monitoring of stress distribution and facilitates non-invasive detection of pressure variations within dynamic systems. As such, piezochromism has found widespread use in structural health monitoring, anti-counterfeiting technologies, and an emerging class of mechanically responsive nano-optoelectronic devices.

While piezochromism itself is a well-established concept, the integration of nanotechnology has significantly expanded its scope and potential. Nanomaterials offer distinct advantages over bulk materials for pronounced piezochromic behavior. Their unique characteristics—such as size-dependent optical properties, high surface-to-volume ratios, defect engineerability, quan-tum confinement, and tunable structures—unlock exceptional sensitivity and novel properties [4,5]. At the nanoscale, mechanical forces induce significant modifications to the electronic and structural properties of materials—such as shifts in electronic band structures, molecular packing, and crystal field environments—that result in controllable, reversible color transitions with unparalleled sensitivity [6]. These force-driven nanoscale modulations manifest as measurable optical responses—including photoluminescence shifts, plasmonic resonance tuning, and deformation-induced structural color variations—thus closely linking piezochromism with the principles of modern nano-optics and nanophotonics. These characteristics make nanomaterials particularly suited for applications requiring high precision and adaptability, positioning piezochromism as a promising mechanism for next-generation nano-optoelectronic and mechano-photonic systems.

Piezochromic nanomaterials also show promise for photovoltaics (PV). Stresses from thermal expansion mismatch, lattice strain, ion migration, or grain-boundary stress mismatch offer an impurity-free method to tune absorber bandgaps, defect energetics, and carrier lifetimes [7,8,9]. These stresses primarily arise from intrinsic sources such as residual lattice strain, which often manifests as in-plane strains between 0.5% and 1.0% in perovskite thin films and corresponds to stresses exceeding 50 MPa [10]. Furthermore, localized stress concentrations at grain boundaries can reach significantly higher levels, with typical values ranging from 300 to 570 MPa [11]. By establishing a direct conceptual bridge between piezochromism and solar-energy conversion and extending the relevance of mechanically responsive nanomaterials to next-generation photovoltaic device engineering.

**Figure 1:**
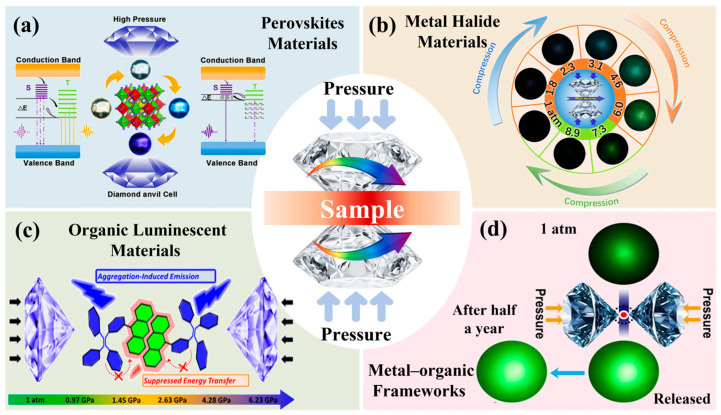
Representative material systems exhibiting piezochromic behavior. (**a**) Perovskite materials. Reproduced with permission [20]. Copyright 2021, American Chemical Society. (**b**) Metal halide materials. Reproduced with permission [21]. Copyright 2022, American Chemical Society. (**c**) Organic luminescent materials. Reproduced with permission [22]. Copyright 2020, American Chemical Society. (**d**) Metal–organic frameworks. Reproduced with permission [23]. Copyright 2022, John Wiley and Sons, Ltd.

Recent advancements have introduced a variety of nanomaterial systems that are particularly well-suited for piezochromic applications. As shown in Figure 1 perovskite materials, metal halide materials, organic luminescent materials and metal–organic frameworks (MOFs) have emerged as key platforms for achieving high-performance piezochromism. Inorganic core–shell nanocrystals, such as InP/ZnSe and InP/ZnS, demonstrate remarkable piezochromic luminescence, attributed to pressure-modulated exciton-phonon coupling and interfacial strain effects [12,13,14]. Similarly, all-inorganic perovskites, such as Cs_3_Cu_2_Cl_5_ nanocrystals, exhibit an exceptional piezochromic range driven by pressure-induced distortions in self-trapped exciton emissions [13]. MOFs, with their porous structures and responsive host-guest interactions, contribute by enhancing the stability of pressure-sensitive emissions [15], particularly when encapsulating perovskite QDs. Organic-inorganic hybrid materials, including molecularly engineered polymers and layer-by-layer assemblies, exploit synergistic interactions to amplify color contrast and pressure sensitivity, enabling further tunability of their mechano-optical responses [16,17,18]. Beyond these active responsive components, the broader landscape of solar cell engineering relies heavily on binary oxide ceramics—including TiO_2_, ZnO, Al_2_O_3_, SiO_2_, CeO_2_, Fe_2_O_3_, and WO_3_—which serve as indispensable charge transport layers, buffer layers, or structural scaffolds. These oxides play a dual role in piezochromic photovoltaic devices by facilitating efficient carrier extraction and acting as strain-mediating interfaces that can modulate the mechanical response of the primary absorber layers [19]. Collectively, these material innovations highlight the critical role of nanoscale structural engineering in enabling mechanically tunable optoelectronic functionality, with direct implications for advanced photoelectric devices and strain-engineered solar cells.

This review provides a comprehensive overview of recent progress in piezochromic nanomaterials, with particular emphasis on their mechanical–optical coupling mechanisms, material platforms, and emerging optoelectronic and photovoltaic applications. We begin by outlining the fundamental principles of piezochromism, detailing how pressure-induced lattice distortion, excitonic reconfiguration, and molecular rearrangement give rise to tunable optical responses. We then summarize the intrinsic characteristics of piezochromic behavior and the extrinsic factors that modulate it, such as particle geometry, surface chemistry, and environmental conditions. Key material classes—including perovskites, metal halides, organic luminogens, and metal–organic frameworks—are systematically examined, highlighting how their structural motifs and nanoscale characteristics dictate pressure sensitivity, spectral tunability, and reversibility. Building on these foundations, we highlight the growing applicability of piezochromic nanomaterials in mechanically reconfigurable optoelectronic platforms and strain-adaptive photovoltaic systems. Finally, we identify the central challenges and research opportunities required to translate piezochromic mechanisms into practical solar-energy technologies, particularly in improving stability, strain transfer efficiency, and device integration.

## 2. Fundamentals of Piezochromism

### 2.1. Mechanisms of Piezochromism at the Nanoscale

**Figure 2:**
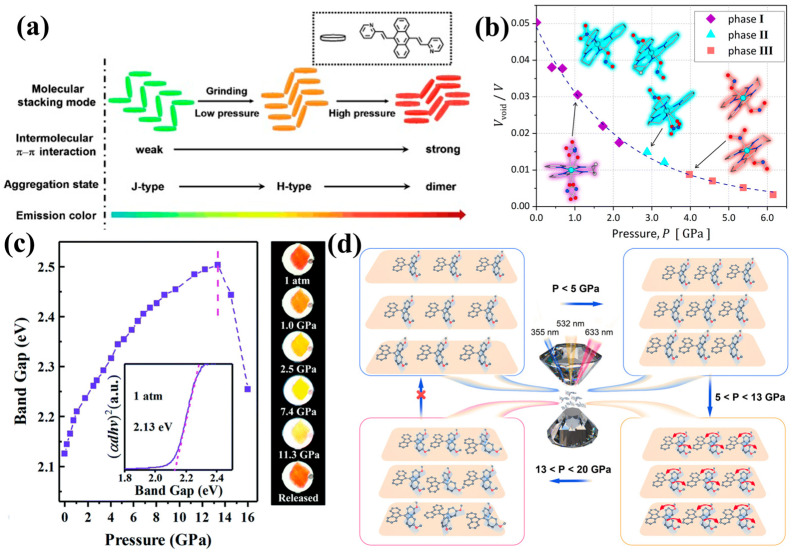
Schematic classification of piezochromic mechanisms. (**a**) Stacking modes and corresponding emission colors for the various molecular aggregation states in BP2VA powder. Reproduced with permission [28]. Copyright 2012, John Wiley and Sons, Ltd. (**b**) Pressure-induced nitrite ligand phase transitions accompanied by a piezochromic effect. Reproduced with permission [29]. Copyright 2024, Royal Society of Chemistry. (**c**) Bandgap and color evolution of the InP/ZnS core/shell NCs at high pressure. Reproduced with permission [14]. Copyright 2020, Royal Society of Chemistry. (**d**) Schematic of FTPE crystal structural evolution under high pressure via multiple excitation channels. Reproduced with permission [30]. Copyright 2022, Springer Nature.

Piezochromism, a subset of mechanochromism, refers to the reversible change in a material’s optical properties—primarily its color or spectral characteristics—upon the application of mechanical pressure. This phenomenon arises from pressure-induced modifications to a material’s electronic structure, crystal lattice, or molecular arrangement [6]. When pressure is applied, the resulting stress and strain alter atomic and molecular distances, shifting electron energy levels and leading to changes in absorption and emission spectra. These shifts, perceived as color changes, typically occur in the visible or near-infrared regions [24,25]. Nanomaterials, with their high surface-to-volume ratios and quantum confinement effects, experience amplified piezochromic responses. The coupling of applied strain with various bonding interactions [26], such as ionic, covalent, and van der Waals forces [27], leads to changes in optical properties that depend on factors such as crystal structure, strain magnitude, surface defects, and chemical environment. Following these fundamental principles, the piezochromic response can originate from several distinct pressure-dependent pathways, each governed by the intrinsic structure and bonding characteristics of materials, as shown in Figure 2.

#### 2.1.1. Molecular-Based Mechanisms

Modulation in organic molecular crystals, dyes, polymers, and pressure can significantly enhance intermolecular interactions such as π-π stacking and hydrogen bonding. Mechanical compression reorganizes intermolecular packing, which alters the collective electronic state and produces distinct changes in the emission spectrum [28]. For flexible organic luminogens, applied pressure induces conformational adjustments, most notably a tendency toward more planar geometries—which suppress intramolecular motions and strengthen π-conjugation. This pressure-driven planarization alters the electronic environment of the emissive states, giving rise to bathochromic shifts in absorption and photoluminescence, as observed in aggregation induced emission (AIE)-active molecules [30].

The piezochromic response in organic molecular systems primarily originates from pressure-dependent pathways that govern intermolecular bonding and packing characteristics. Under mechanical stress, the reorganization of molecular aggregation states facilitates the redistribution of excitons and the modulation of the electronic environment. These structural transitions frequently promote intermolecular charge transfer (ICT), which alters the collective electronic state and drives the significant optical shifts observed in organic molecular systems [31,32]. Such modifications to the electronic environment under pressure are central to achieving high-sensitivity optical responses in these materials.

At the molecular level, the piezochromic response can also originate from the alteration of covalent bonds and fundamental changes in molecular conformation. These mechanistic pathways involve a pressure–structure–property relationship where the coupling of applied strain with bonding interactions—including covalent and van der Waals forces—leads to a reorganization of the molecular aggregation state. This “activation” of the molecular structure under stress provides the conceptual foundation for tailoring materials for advanced mechano-optical applications [32,33].

#### 2.1.2. Crystal Structure & Defect Engineering

In crystalline materials, applied pressure can induce a structural phase transition, which results in the formation of distinct lattice structures and revised electronic band structures. These transitions often lead to an abrupt and significant change in optical properties. For example, the Di-exo-nitrito [29] nickel(II) complex exhibits multiple phase transitions under pressure, with each phase associated with a unique photoluminescent signature. Similarly, in inorganic semiconductors like quantum dots (QDs), pressure compresses the crystal lattice and modifies the electronic band structure [6]. This lattice compression typically leads to an increase in bandgap energy, causing a blue shift in both absorption and emission spectra, a behavior clearly demonstrated by materials such as InP nanocrystals [14].

The piezochromic behavior of nanomaterials is significantly influenced by their high surface-to-volume ratios and the presence of surface defects. Surface chemistry and functionalization represent a crucial dimension in determining how these materials respond to mechanical stimuli. Because surface atoms have reduced coordination and altered bonding environments, they are far more sensitive to external forces than bulk atoms. Consequently, nanomaterials with abundant surface defects, such as vacancies or dislocations, or those with specific tailored surface moieties, exhibit modulated pressure-induced optical responses [12,14,33]. Furthermore, ligand modifications on nanocrystals can change how pressure is transmitted to the core and how the electronic structure responds, thereby influencing the threshold and magnitude of the color change [26].

#### 2.1.3. Plasmonic and Exciton Effects

The morphology of nanoparticles—whether they are spherical, rod-like, plate-like, or other anisotropic shapes—critically determines how mechanical strain is distributed within the structure. This distribution directly influences the degree and direction of the resulting spectral shift. As the size of a nanomaterial decreases, its surface-to-volume ratio increases, which often leads to amplified piezochromic responses. Smaller particles experience greater lattice deformation under pressure, which enhances the optical shift relative to their larger counterparts. Studies on semiconductor nanocrystals have further demonstrated that this behavior is highly dependent on both nanoscale geometry and surface chemistry, as ligands can modulate how external pressure is transmitted to the core [12,14].

In inorganic semiconductors such as quantum dots (QDs), applied pressure compresses the crystal lattice, which in turn modifies the electronic band structure. This compression typically leads to an increase in bandgap energy, resulting in a characteristic blue shift in both absorption and emission spectra. These pressure-induced changes alter the electronic environment of the emissive states and modulate the collective electronic state of the material. The coupling of applied strain with various bonding interactions leads to changes in optical properties that are fundamentally governed by the crystal structure and the magnitude of the applied strain [34].

### 2.2. Characteristics of Piezochromic Effect

The characteristics of the piezochromic effect are defined by the quantitative relationships and dynamic behaviors governing pressure-dependent optical responses. To provide a systematic comparison across different material classes, the primary mechanisms, structural responses, and characteristic behaviors of piezochromic materials are summarized in Table 1 For practical applications—particularly in sensing and optical modulation—key descriptors include sensitivity, linearity, reversibility, and hysteresis. The correlation between applied pressure and the resulting spectral shift is central to evaluating performance, and linear pressure–spectral shift relationships are preferred for predictable calibration [35]. Sensitivity varies widely across material systems, with some exhibiting modest shifts and others showing exceptionally large contrasts. Certain nanomaterials also maintain reversible piezochromic behavior over broad pressure ranges extending from ambient conditions to several gigapascals [36].

Reversibility plays a critical role in determining the usability of piezochromic materials, yet its manifestation depends strongly on the underlying mechanism. Materials governed predominantly by weak intermolecular interactions often display highly reversible responses because the structural perturbations induced by pressure are elastic and readily recoverable [44]. In contrast, systems that undergo significant structural reorganization—such as covalent framework distortion or pressure-induced phase transitions—are more prone to hysteresis. Hysteresis occurs when the optical properties during decompression do not retrace the compression pathway, reflecting kinetic barriers or energy dissipation processes. Although hysteresis is sometimes viewed as an indication of incomplete recovery, it can also be exploited in optical memory or data-retention applications [27]. Thus, reversibility and hysteresis are not mutually exclusive but instead represent different outcomes within the broader dynamic response of piezochromic materials.

From a functional perspective, piezochromic responses can be broadly classified as reversible or irreversible. Reversible piezochromism arises from non-destructive modulations—such as changes in molecular packing, excited-state electronic structure, or coordination environment—that fully return to the initial optical state upon pressure release. Irreversible piezochromism, on the other hand, is associated with permanent structural transformations, including bond rearrangements, phase transitions with high kinetic barriers, or pressure-induced chemical changes. While reversible responses are preferred for dynamic sensing and real-time monitoring, irreversible responses may be advantageous for recording, authentication, or anticounterfeiting functions. The interplay among sensitivity, linearity, reversibility, and hysteresis forms the basis for evaluating piezochromic materials. These metrics establish design principles for strain-mediated bandgap tuning, defect modulation, and stability engineering in emerging photovoltaics.

### 2.3. Factors Affecting Piezochromism

Several factors influence the magnitude and nature of piezochromism in nanomaterials. One of the primary factors is particle size and shape. As the size of a nanomaterial decreases, its surface-to-volume ratio increases, which often leads to amplified piezochromic responses. For example, smaller particles experience greater lattice deformation under pressure, enhancing the optical shift relative to larger counterparts. In addition, nanoparticle morphology—including spherical, rod-like, and plate-like shapes—dictates internal strain distribution, governing both the magnitude and direction of spectral shifts [26]. Studies on semiconductor nanocrystals under pressure have shown a pronounced size- and ligand-dependent piezochromic behavior, underscoring the critical role of nanoscale geometry and surface chemistry [12].

Surface chemistry and functionalization represent another crucial dimension in determining piezochromic behavior. Nanomaterials with abundant surface defects, functional groups or adsorbed molecules respond differently to mechanical stimuli compared with defect-free or passivated systems. Surface atoms are more sensitive to external forces because of their reduced coordination and altered bonding environment, so the presence of defects or tailored surface moieties can significantly modulate the material’s pressure-induced optical response. For instance, ligand modification on nanocrystals may change how pressure is transmitted to the core and how the electronic structure responds, thus influencing both the threshold and magnitude of the color change.

Environmental conditions such as temperature and humidity also affect piezochromic performance by altering the mechanical and chemical environment of the material [34]. Temperature variations may change the elasticity or modulus of nanostructures, thereby modifying how strain is accommodated under pressure. Meanwhile, humidity can influence intermolecular or ion–molecule interactions, altering the baseline optical state and the sensitivity to pressure. Consequently, when designing piezochromic nanomaterials for practical applications, it is essential to consider not only intrinsic material properties but also the operational environment, which may shift the observed response and stability of the device.

## 3. Nanomaterials for Piezochromic Applications

### 3.1. Perovskite Materials

**Figure 3:**
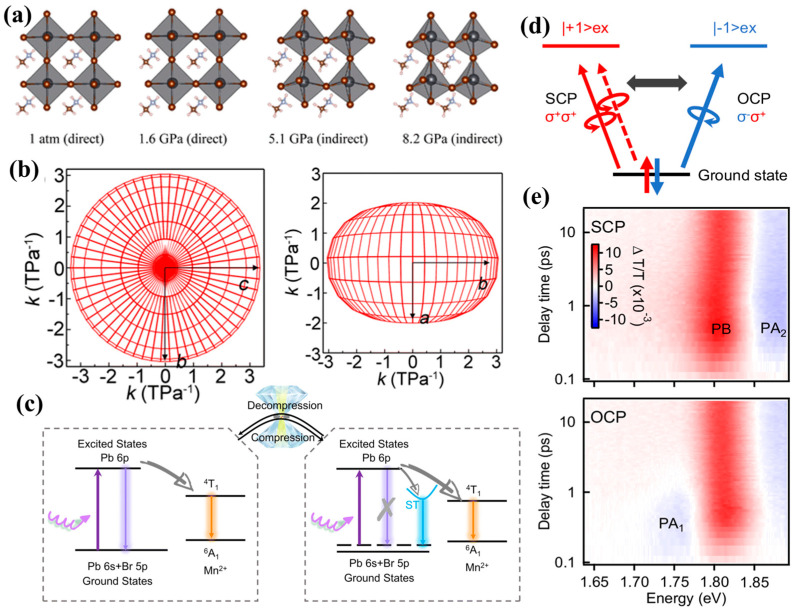
(**a**) Evolution of MAPbBr_3_ structures under four critical pressure values. Reproduced with permission [47]. Copyright 2018, American Chemical Society. (**b**) Compressibility indicatrix in the bc and ab planes of Mn-doped (PEA)_2_PbBr_4_ NCs. Reproduced with permission [37]. Copyright 2023, Royal Society of Chemistry. (**c**) Configuration coordinate models for Mn doped (PEA)_2_PbBr_4_ under ambient conditions and high pressure. Reproduced with permission [37]. Copyright 2023, Royal Society of Chemistry. (**d**,**e**) Spin-resolved transient absorption spectroscopy of (PEA)_2_(MA)Sn_2_I_7_ thin flake at room temperature. Reproduced with permission [45], Copyright 2024, Springer Nature.

The piezochromic response in perovskites is primarily governed by their lattice-driven structural flexibility, where the modulation of ABX3 frameworks—such as octahedral tilting and bond contraction—directly alters the electronic band structure [45,46]. As shown in Figure 3a, even modest mechanical compression can induce tilting, distortion, or contraction of octahedra [47], leading to significant changes in bandgap energy and photoluminescence properties. At the nanoscale, quantum confinement further enhances the piezochromic sensitivity of perovskite nanocrystals [48]. The combination of soft lattice dynamics and large surface-to-volume ratios enables efficient transduction of mechanical strain into spectral shifts, including reversible photoluminescence redshifts or blueshifts depending on the dominant structural deformation mode. Two-dimensional (2D) halide perovskites also exhibit unique piezochromic signatures due to their anisotropic compressibility (Figure 3b,c) and localized excitons (Figure 3d,e), making them suitable for opto-pressure sensing [37,45].

**Figure 4:**
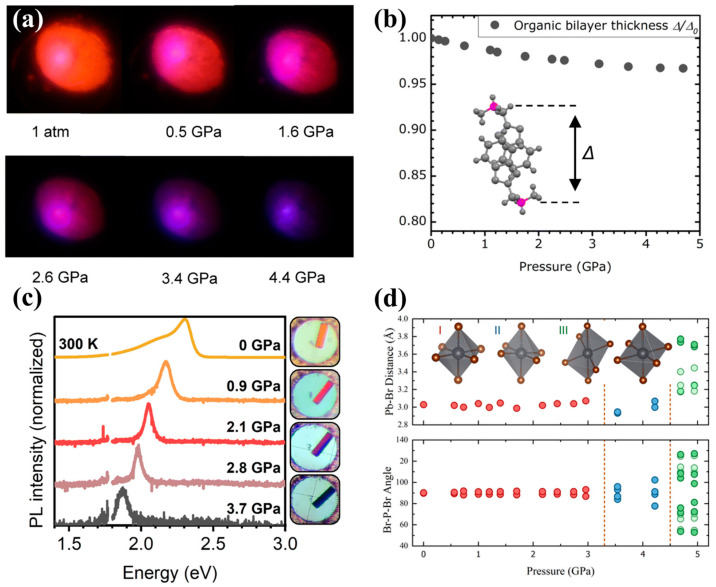
(**a**) PL micrographs upon compression of Mn-doped (PEA)_2_PbBr_4_ NCs under high pressure. Reproduced with permission [37]. Copyright 2023, Royal Society of Chemistry. (**b**) Thickness of the organic bilayers as a function of pressure and (**c**) PL emission shift in a (BTa)_2_PbI_4_ single crystal with pressure, causing the crystal to change color. Reproduced with permission [38] Copyright 2025, Royal Society of Chemistry. (**d**) Pb–Br distance and Br–Pb–Br angle of the PbBr_6_ octahedron as a function of the hydrostatic pressure. Reproduced with permission [49]. Copyright 2022, American Chemical Society.

Building on these fundamental lattice-driven piezochromic behaviors, a wide range of perovskite-related nanomaterials exhibit distinct optical responses under pressure, reflecting the diversity of their structural motifs. Mn^2+^-doped 2D (PEA)_2_PbBr_4_ nanocrystals exhibit a different type of modulation, undergoing a smooth color evolution from orange to blue-violet within 0–5 GPa as their bandgap decreases from 2.3 eV to 1.9 eV. The presence of Mn^2+^ introduces local lattice distortions, and additional compression further twists the PbBr_6_ octahedra, producing continuous blueshifts in emission (Figure 4a) [37]. Further insights arise from layered hybrid perovskites and 0D halide derivatives, which highlight additional pressure-response pathways. Layered perovskites incorporating cross-linked organic bilayers exhibit fully reversible piezochromism across the visible spectrum. Their color tunability is driven by significant compression of the organic interlayers, which reduces interlayer spacing and strengthens electron–phonon coupling (Figure 4b,c) [38]. Meanwhile, 0D Cs_4_PbBr_6_ nanocrystals undergo luminescence transition within 0–5 GPa, arising from shortened Pb–Br bond lengths and increased local rigidity that reduce exciton binding energy and alter emission pathways (Figure 4d) [49]. These varied pressure-modulated behaviors across different structural families illustrate the versatility of perovskite-based systems and underscore their potential in pressure sensing, optical anti-counterfeiting, and mechanically controlled information storage.

These diverse pressure-responsive behaviors highlight the structural flexibility that make perovskites exceptionally versatile piezochromic materials. As high-pressure studies continue to clarify how lattice compression and octahedral tilting govern charge transport and phase stability of perovskite materials, a general rule-of-thumb has emerged: the bandgap trajectory in these frameworks is determined by the competition between bond contraction and structural distortion. While initial compression shortens metal–halide bonds to increase orbital overlap, subsequent pressure-induced octahedral tilting or twisting often reduces this overlap, ultimately triggering a blueshift or a discontinuous spectral jump during phase transitions. Continued advances in compositional tuning, dimensional engineering, and mechanical robustness will further accelerate the integration of piezochromic principles into next-generation high-efficiency, durable perovskite solar technologies.

### 3.2. Metal Halide Materials

**Figure 5:**
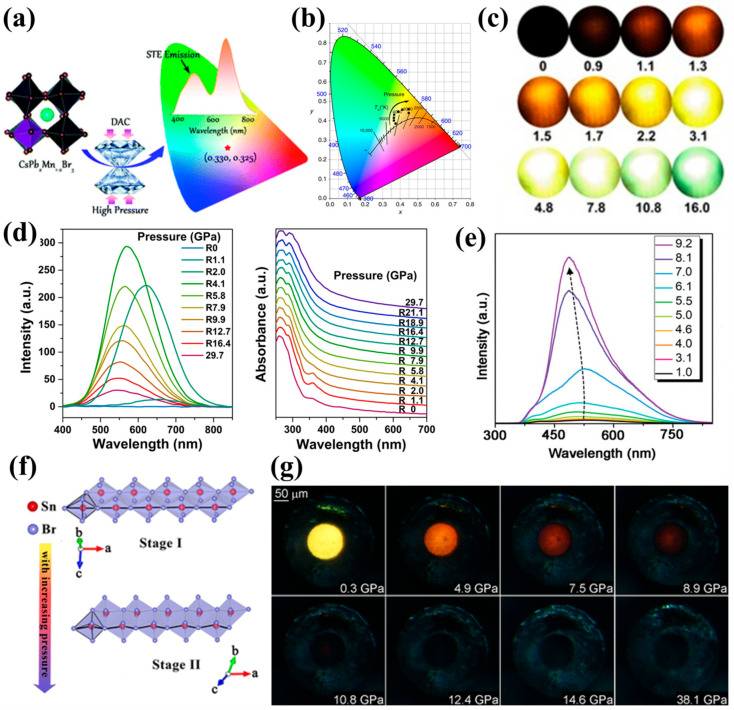
(**a**) Self-trapped exciton emission and piezochromism in conventional 3D lead bromide perovskite nanocrystals under high pressure. Reproduced with permission [39]. Copyright 2021, Royal Society of Chemistry. (**b**) Pressure-dependent Cs_4_PbBr_6_ NC chromaticity coordinates of the emissions. Reproduced with permission [40]. Copyright 2018, Springer Nature. (**c**) Optical micrographs and (**d**) spectra of the pressure-induced changes in Cs_3_Sb_2_Br_9_ QDs. Reproduced with permission [41]. Copyright 2024, Taylor and Francis, Ltd. (**e**) High-pressure PL evolution of Cs_3_Cu2I_5_ NCs upon compression to 9.2 GPa. Reproduced with permission [53]. Copyright 2022, Wiley-VCH Verlag. (**f**) Transformation of octahedral chain views of C_4_N_2_H_14_SnBr_4_ under high pressure. Reproduced with permission [54]. Copyright 2019, American Chemical Society. (**g**) Optical micrographs in a diamond anvil cell, displaying piezochromic transitions from translucent yellow to red to opaque black. Reproduced with permission [25]. Copyright 2015, American Chemical Society.

Metal halide nanomaterials exhibit prominent piezochromic behaviors among halide-based systems, largely due to their deformable coordination polyhedra and localized excitonic states. While sharing similar chemical components with perovskites, these systems are distinguished by their exciton-dominated mechanisms. Their piezochromic behavior is fundamentally rooted in the rearrangement of self-trapped exciton (STE) energy levels within isolated coordination polyhedra. Mechanical compression reshapes metal–halide octahedra such as [PbBr_6_]^4−^, [SnBr_6_]^4−^, and [CuCl_6_]^4−^ through bond length contraction, angular tilting, or octahedral twisting, which collectively modulate the bandgap, exciton binding energies, and radiative recombination pathways (Figure 5a) [39,50,51,52].

Among the various structural families, zero-dimensional metal halides stand out due to their molecular-like emissive units and strong exciton localization. Cs_4_PbBr_6_ nanocrystals, for example, display pressure-induced emission (PIE), evolving from non-emissive to intense green emission under 0–5 GPa as distortion of the isolated [PbBr6]^4−^ clusters increases local lattice rigidity and strengthens STE confinement (Figure 5b) [40]. Similar systems such as Cs_3_Pb_2_Br_9_ and Cs_3_Cu_2_I_5_ exhibit even larger spectral transformations, showing a pronounced piezochromic evolution over 0–16 GPa, with its emission color shifting dramatically from the initial red to a vivid lime-green (Figure 5c). Concurrently, Cs_3_Pb_2_Br_9_ demonstrates exceptional reversibility and structural stability during the decompression process (Figure 5d) [41]. Cs_3_Cu_2_I_5_ displays pressure-sensitive STE emission modulation, where the primary 440 nm band undergoes a gradual blueshift and a secondary 550 nm band is progressively enhanced up to 1.0 GPa, resulting in clear pressure-induced color changes (Figure 5e) [53].

Depending on the extent and reversibility of such structural or interfacial changes, metal halides may display reversible redshift/blueshift behaviors or retain high-energy STE states after decompression, giving rise to pronounced memory effects. One-dimensional organic tin halides offer another class of strongly pressure-responsive materials. Compounds such as C_4_N_2_H_14_SnBr_4_ display pressure-activated photoluminescence as structural rearrangements of the [SnBr_6_]^4−^ octahedra enhance the STE transition dipole moment and binding strength, producing marked blueshifts and emission intensification (Figure 5f) [54]. Two-dimensional halide frameworks also exhibit strong piezochromism due to their inherently anisotropic compressibility. Layered hybrid perovskites containing cross-linked organic bilayers show large reductions in interlayer spacing above 10 GPa, intensifying electron–phonon interactions and enabling reversible color tuning across the entire visible range [41]. By contrast, Cu–Cl bilayer halides undergo two pressure-induced phase transitions between 4 and 8 GPa, transitioning from yellow to red to black (Figure 5g) while simultaneously exhibiting enhanced electrical conductivity. The suppression of Cu^2+^ Jahn–Teller distortion under compression narrows the bandgap, giving rise to dramatic halochromic effects [25].

Metal halide nanomaterials, with their soft ionic lattices and strongly localized excitonic states, provide a uniquely tunable platform for pressure-responsive optical behavior. In these localized systems, a distinct rule-of-thumb applies where the optical response is dominated by STE dynamics. Specifically, pressure increases lattice rigidity and distorts isolated polyhedra, which elevates the energy levels of STE states and favors a continuous blueshift in emission. Such reversible piezochromic shifts can translate to capabilities desirable for solar energy conversion, including bandgap modulation toward optimal absorption, defect state reorganization that suppresses non-radiative losses, and the stabilization of photoactive phases. As high-pressure research clarifies strain-governed carrier dynamics and stability, metal halides emerge as candidates for strain-engineered absorbers and self-monitoring modules, advancing next-generation solar technologies.

### 3.3. Organic Luminescent Materials

Organic luminescent materials represent a highly versatile and tunable class of piezochromic systems owing to their flexible molecular backbones, rich excited-state dynamics, and diverse noncovalent interaction networks. Unlike inorganic halides, organic luminogens exhibit piezochromism through changes in intermolecular packing, molecular conformation, and intramolecular charge transfer (ICT) states. Mechanical pressure can alter π–π stacking distances, hydrogen-bonding patterns, or donor–acceptor (D–A) geometries, leading to substantial shifts in emission color and intensity. In many cases, pressure-induced restriction of intramolecular motion (RIM) enhances radiative decay pathways, while planarization of conjugated backbones strengthens π-conjugation and adjusts the HOMO–LUMO gap.

**Figure 6:**
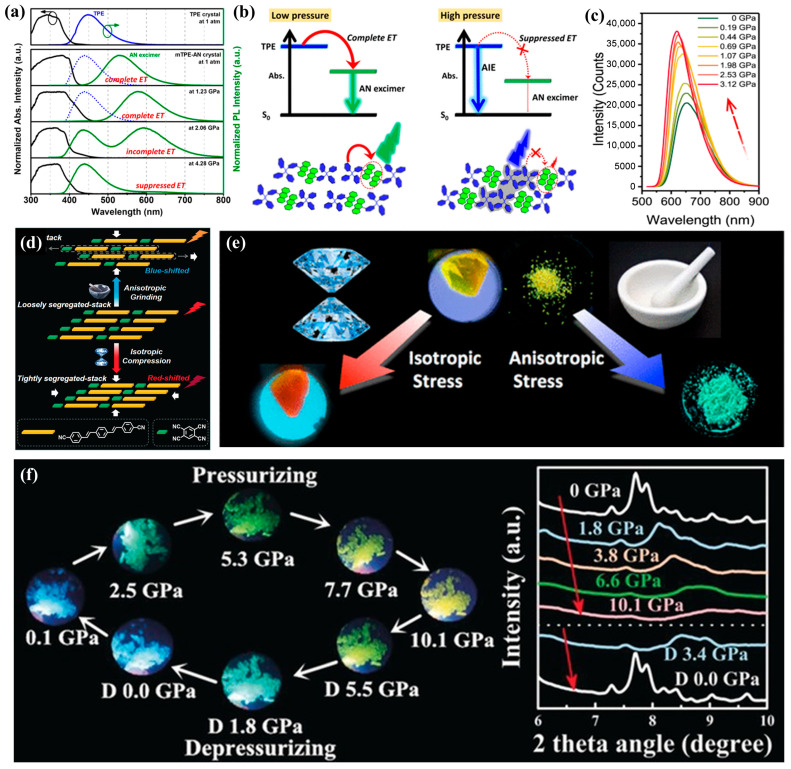
(**a**) Normalized absorption and emission spectra of TPE crystals at 1 atm and mTPE-AN crystals at 1 atm 4.28 GPa. (**b**) Schematic diagram of pressure-induced emission blue-shift and enhancement in mTPE-AN crystals. Reproduced with permission [22]. Copyright 2020, American Chemical Society. (**c**) PL spectra of PTCs–THF up to 3.12 GPa. Reproduced with permission [17]. Copyright 2021, Springer Nature. (**d**) Transition modeling schematic of molecular stacking mode in CT-R cocrystal under different mechanical force stimuli. Reproduced with permission [57]. Copyright 2018, John Wiley and Sons, Ltd. (**e**) Distinct responses to mechanical grinding and hydrostatic pressure in luminescent chromism of tetrathiazolylthiophene. Reproduced with permission [58]. Copyright 2013, American Chemical Society. (**f**) Micrographs and in situ ADXRD patterns of the piezochromic material containing tetraphenylethylene at pressures from 0 to 10.1 GPa. Reproduced with permission [35].

Aggregation-induced emission luminogens represent one of the most pressure-responsive categories. In the crystal of 9-(3-(1,2,2-triphenylvinyl)phenyl)anthracene, mechanical compression produces an unusual blueshift accompanied by pronounced luminescence enhancement in the 1.23–4.28 GPa range (Figure 6a). This behavior arises from the synergistic interplay between the aggregation-induced emission effect of the TPE unit and the suppression of energy-transfer pathways under pressure. Another representative AIE-based system is the TPE–AN co-crystal, which undergoes a gradual color evolution from greenish-yellow to blue as pressure increases. The transition is attributed to pressure-enhanced molecular packing that strengthens the AIE effect and simultaneously inhibits intermolecular energy transfer (Figure 6b) [22,55]. Recent reviews highlight that AIE luminogens, with their inherently twisted conformations, readily undergo pressure-induced planarization. This structural transition effectively reinforces π-conjugation and modulates excited-state relaxation, resulting in large bathochromic or hypsochromic shifts [56].

Donor–acceptor (D–A) co-crystals such as perylene–TCNB normally exhibit conventional redshifts and fluorescence quenching under hydrostatic compression. However, introducing THF molecules into the lattice subtly adjusts hydrogen bonding and D–A conformations, leading to an anomalous blueshift and enhanced emission when pressed to 3 GPa (Figure 6c) [17]. A related D–A co-crystal system shows a continuous redshift during isotropic compression, whereas anisotropic grinding triggers a blueshift, reflecting the competition between tightly packed structures and shear-induced molecular reorganization (Figure 6d) [57]. Moreover, benzothiazole–amide BF_2_ complexes featuring helical AIE backbones exhibit distinct redshift or blueshift behaviors depending on pressure, governed by the balance between π–π interactions and intramolecular charge transfer (ICT) modulation [27]. These observations further validate the strong coupling between D–A architecture, intermolecular packing, and pressure-dependent optical responses.

In purely π-stacked systems, tetrathiazole–thiophene derivatives display a blueshift under anisotropic shear and a redshift under hydrostatic compression (Figure 6e). The contrasting responses originate from the suppression or enhancement of excimer formation: shear disrupts π-stacking and weakens excimer stabilization, whereas uniform compression shortens π–π distances and promotes excimer-mediated emission. These cases demonstrate that π-stacking topology and compressibility critically dictate the direction and magnitude of piezochromic shifts in organic crystals [58]. Additionally, a tetraphenylethylene-based piezochromic material exhibits observable fluorescence throughout a pressure range of 0.0–10.1 GPa, demonstrating a linear correlation between the applied pressure and the peak emission wavelength alongside excellent reversibility (Figure 6f) [35].

Organic luminescent materials offer an exceptionally broad design space for piezochromic engineering due to their structural softness, conformational flexibility, and tunable excited-state properties. Whether through restriction of intramolecular motion, modulation of charge transfer states, or compression of π-stacked frameworks, these materials enable large, reversible, and highly designable mechanochromic responses. Their molecular versatility positions them as strong candidates for next-generation pressure sensors, optical encryption technologies, and flexible optoelectronic devices.

### 3.4. Metal–Organic Frameworks

Metal–organic frameworks (MOFs) offer a unique platform for piezochromic applications owing to their structurally flexible coordination networks, intrinsic porosity, and highly tunable host–guest interactions [59,60,61]. Unlike purely inorganic semiconductors or organic luminogens, MOFs integrate metal nodes with organic linkers, allowing mechanical pressure to modulate both coordination geometry and ligand electronic structure. Compression can induce changes such as metal–ligand bond contraction, linker rotation, framework breathing, or pore collapse, all of which influence excitonic processes, charge transfer pathways, and photoluminescence behavior. In many luminescent MOFs, pressure enhances metal-centered or ligand-centered excitations by altering frontier orbital overlap, while in host–guest systems, pressure-controlled confinement modifies guest emission or promotes energy transfer between the two components. The inherent flexibility of MOFs thus provides multiple stimuli-responsive pathways for optical modulation.

**Figure 7:**
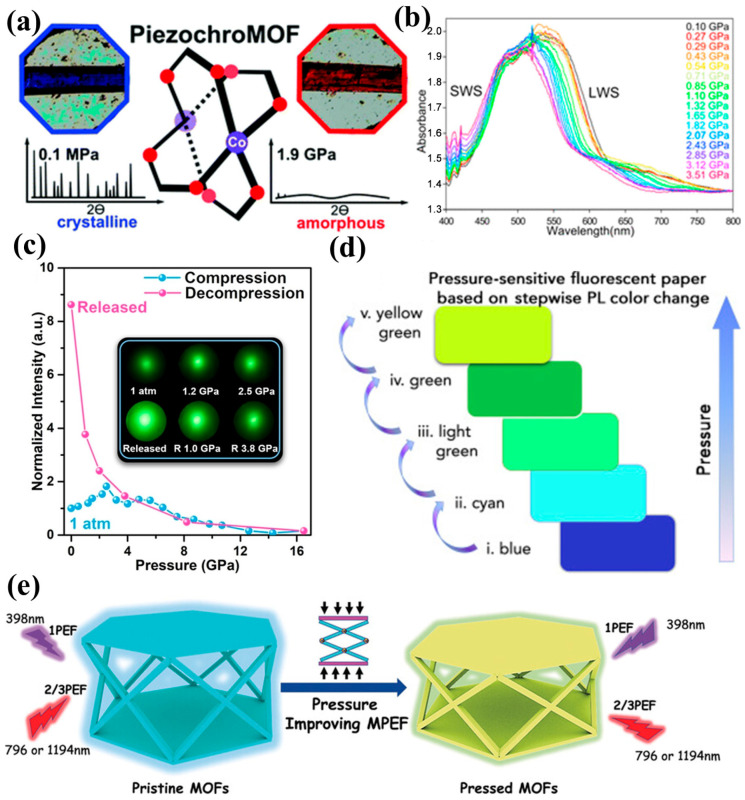
(**a**) Reversible pressure pre-amorphization of a piezochromic metal–organic framework. Reproduced with permission [42]. Copyright 2017, Royal Society of Chemistry. (**b**) Selected high-pressure VIS spectra of AMU-1. The pressure-induced changes in the spectrum described by the shifts of the long-wavelength slope (LWS) and short-wavelength slope (SWS) of the overlapped bands. Reproduced with permission [62]. Copyright 2016, American Chemical Society. (**c**) PL intensity of Tb(BTC)(H_2_O)_6_ as a function of pressure, and the inset is the trend of the PL micrographs at selected pressures. Reproduced with permission [23]. Copyright 2021, John Wiley and Sons, Ltd. (**d**) Pressure-sensitive fluorescent paperbased on stepwise PL color change. Reproduced with permission [63]. Copyright 2018, Elsevier Inc. (**e**) 1/2/3PEF (photon-excited fluorescence) was correlated with pressure-induced fluorochromism in MOFs. Reproduced with permission [43]. Copyright 2019, John Wiley and Sons, Ltd.

Piezochromism in metal–organic frameworks frequently originates from pressure-induced modulation of local coordination environments and framework deformation. Early examples, such as Co_2_(Bdc)_2_Dabco·4DMF·H_2_O, reveal that hydrostatic pressure induces competing distortions in Co^2+^ coordination geometries and Bdc linker conformations, triggering symmetry lowering, pre-amorphization, and pronounced crystal-field reconstruction (Figure 7a). These structural changes strongly modify the d–d transitions of Co^2+^, causing a visually perceptible color evolution from blue to red [42]. Similar coordination-field–driven mechanisms are also observed in the related CoBbcDabcoH_2_O framework, which undergoes a monoclinic-to-triclinic phase transition under modest pressure as linker orientation and water-mediated hydrogen bonding reorganize. In these systems, the strain-induced modulation of Co^2+^ ligand fields produces highly reversible piezochromism (Figure 7b), making such frameworks promising for ultrahigh-precision optical pressure calibration [62]. Beyond transition metal centers, lanthanide MOFs also exhibit pressure-activated optical enhancement. For example, in green-emitting Tb(BTC)(H_2_O)_6_, compression strengthens hydrogen bonding and rigidifies carboxylate–benzene conformations, which facilitates intersystem crossing and markedly enhances ligand-to-metal energy transfer. This results in significant improvements in photoluminescence efficiency, demonstrating that mild structural locking under pressure can effectively optimize LMET pathways (Figure 7c) [23].

At a more global structural level, MOF piezochromism can also arise from pressure-dependent changes in chromophore packing, framework flexibility, and pore contraction. Mixed-linker architectures such as LIFM-66W, built from dual BTB and ETTC chromophores, exhibit ultrasensitive piezofluorochromism in which UV emission is rapidly quenched, while blue emission gradually shifts toward yellow with increasing pressure (Figure 7d). This fine-tunable and orientation-dependent response reflects the anisotropic deformation of the framework and the differential compression of co-aligned chromophores [63]. More complex optical responses appear in MOFs that combine multiphoton absorption with piezochromism. Frameworks such as LIFM-114, featuring flexible topologies and densely packed chromophores, display simultaneous one-, two-, and three-photon excited fluorescence whose emission shifts from blue to yellow upon compression. Pressure-induced pore contraction enhances π–π interactions and increases the two-photon absorption cross-section by more than an order of magnitude, exemplifying how mechanical stimuli can synergistically amplify upconversion performance. These collective observations highlight that both local coordination deformation and global structural compression—together with linker topology, chromophore orientation, and pore evolution—govern the versatile piezochromic behaviors observed across MOF systems (Figure 7e) [43].

Overall, MOFs represent a structurally versatile and conceptually rich class of piezochromic materials, distinguished by their hybrid inorganic–organic nature and highly modular design. Their tunable porosity, flexible coordination environments, and capacity for host–guest engineering provide multiple pathways for pressure-responsive optical modulation. Continued innovation in linker design, guest encapsulation, and framework mechanics is expected to further broaden the role of MOFs in adaptive photonic devices, multifunctional sensors, and mechanically gated information technologies.

### 3.5. Other Materials

Beyond halide perovskites, metal halides, organic luminogens, and MOFs, several additional classes of nanomaterials exhibit notable piezochromic behaviors enabled by their tunable electronic structures and mechanically responsive bonding environments, including compound semiconductor nanomaterials, transparent conducting oxides (TCOs), organic–inorganic hybrid materials, and carbon-based quantum dots (CDs).

**Figure 8:**
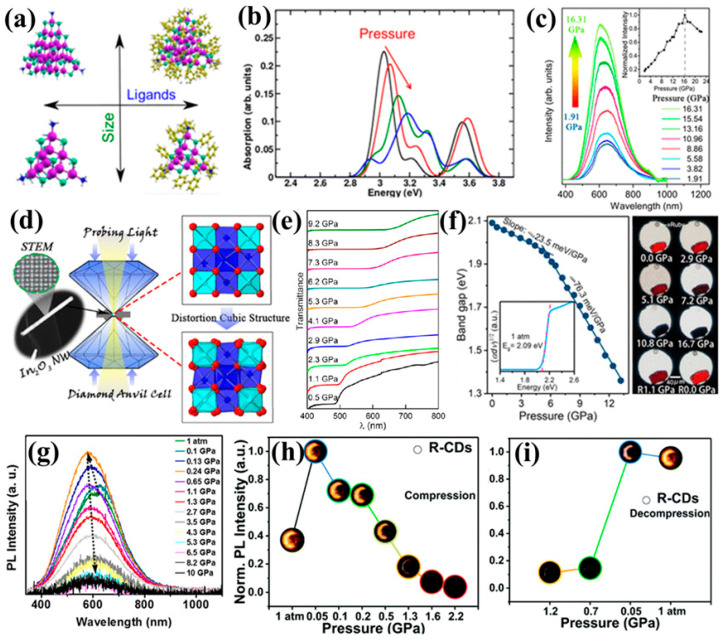
(**a**) Simulated CdS nanocrystal structures. (**b**) TDDFT optical absorption spectra at various pressures. Reproduced with permission [26]. Copyright 2017, American Chemical Society. (**c**) PL spectra of NWs and bulk In_2_O_3_ as a function of pressure. (**d**) Schematic of the cubic crystal bixbyite structure of In_2_O_3_. Reproduced with permission [65]. Copyright 2020, American Chemical Society. (**e**) Transmittance spectra of Py-FeCl_4_ from 0 to 9 GPa during the compression cycle. Reproduced with permission [6]. Copyright 2024, Wiley-VCH. (**f**) Band gap evolution of MA_3_Bi_2_I_9_ upon compression and optical micrographs of MA_3_Bi_2_I_9_ in a diamond anvil cell upon compression. Reproduced with permission [66]. Copyright 2019, American Chemical Society. (**g**) PL spectra of F, N-doped CDs as the pressure increases under ambient conditions. Reproduced with permission [67]. Copyright 2019, John Wiley and Sons, Ltd. (**h**,**i**) PL emissive peak changes in R-CDs with increasing and releasing pressure. Reproduced with permission [68]. Copyright 2019, Royal Society of Chemistry.

Compound semiconductor nanomaterials, including III–V and II–VI systems, exhibit piezochromic behavior primarily through pressure-induced modulation of band structures, exciton localization, and ligand–surface interactions. In Eu-doped GaN, applied pressure enhances bound-exciton localization and suppresses thermal quenching, producing a nearly tenfold increase in Eu^3+^ emission intensity without significant spectral shifts [64]. CdS nanocrystals show more complex responses in which pressure-induced deformation competes with ligand-mediated orbital hybridization; hydrogen-capped nanocrystals typically undergo bandgap widening and blueshift, whereas phenyl-capped ones may show unexpected redshifts due to ligand-induced structural distortion and altered frontier-orbital overlap (Figure 8a,b) [26]. All-inorganic InP/ZnSe nanocrystals display some of the strongest responses, with their luminescence shifting stepwise from red to green and achieving a broad bandgap tunability of 0.46 eV under pressures up to 14.2 GPa. Pressure also drives their assembly from discrete nanocrystals into 2D nanosheets, demonstrating that mechanical stimuli can simultaneously modulate optical transitions and induce stress-driven structural reconfiguration [12].

Transparent conductive oxides also exhibit distinctive piezochromic behaviors driven by pressure-modulated defect states, plasmonic interactions, and nanoscale confinement. ISOH-type inorganic nanopowders composed of nanometer-scale voids display unusually high-pressure sensitivity, showing color changes at ultralow pressures of only 0.002–0.01 GPa. This response is attributed to uniform nanoscale microvoids that strongly modify quantum confinement and surface-plasmon interactions between conductive ITO domains and insulating hydroxide regions [4]. In contrast, individual In_2_O_3_ nanowires exhibit pronounced pressure-induced photoluminescence enhancement, increasing emission intensity by more than fourfold. DFT analysis links this behavior to pressure-driven overlap between oxygen-defect states and the conduction band minimum—an effect absent in bulk In_2_O_3_. Experimental measurements show discontinuities in PL and absorption near 16 GPa, consistent with Rietveld-identified distortions of InO_6_ octahedra and trigonal prisms (Figure 8c,d). Notably, the optical transmittance of In_2_O_3_ nanowires partially recovers upon decompression and can even surpass the original baseline after high-pressure cycling, underscoring the important role of local lattice distortion and defect reorganization in enabling reversible or enhanced piezochromic responses in TCO nanostructures [65].

Organic–inorganic hybrid systems represent another important class of piezochromic materials, where pressure-responsive optical behaviors arise from the coupled deformation of inorganic frameworks and electronically active organic moieties. A prototypical example is 2,4,6-triphenylpyrylium tetrachloroferrate (Py–FeCl_4_), which exhibits fully reversible multicolor switching over 0–9 GPa. Spectroscopic analyses indicate that this behavior is not accompanied by structural phase transitions; instead, the pronounced bandgap changes originate from pressure-modulated electron transfer between the FeCl_4_^−^ inorganic anion and the pyrylium organic cation, demonstrating a purely electronic piezochromic mechanism (Figure 8e) [6]. Hybrid halide compounds also show striking responsiveness, as seen in (CH_3_NH_3_)_3_Bi_2_I_9_, which undergoes pressure-induced PL enhancement, bandgap narrowing, and dramatic color evolution from transparent red to opaque black. These changes correlate with a hexagonal-to-monoclinic phase transition near 5 GPa and reversible amorphization at 29 GPa, accompanied by the ordering–disordering transition of the MA^+^ cations and significant conductivity enhancement (Figure 8f) [66]. Beyond salt-type hybrids, few-layer g-C_3_N_4_ demonstrates tunable emission from blue to yellow and anomalous PL enhancement at very low pressures, driven by strengthened interlayer coupling that modulates electron–hole separation. At higher pressures, an interlayer stacking transition reduces compressibility, leading to PL broadening and quenching [69]. Collectively, these examples underline the rich piezochromic tunability accessible in hybrid systems, where synergistic organic–inorganic interactions enable large spectral shifts, reversible color cycling, and pressure-activated optoelectronic transitions with potential for sensing, optical switching, and photonic memory devices.

Carbon-based quantum dots (CDs) represent a distinctive class of piezochromic nanomaterials owing to their flexible carbon frameworks, abundant surface states, and pressure-responsive aggregation behavior. Fluorine–nitrogen co-doped CDs exhibit photoactivated fluorescence enhancement and a reversible piezochromic response from 1 atm to 10 GPa, where external pressure triggers aggregation-induced emission at low pressure and modulates surface-state energetics at higher loads (Figure 8g) [67]. Red-emissive CDs show similar tunability: their emission shifts toward the near-infrared with increasing concentration, and applied pressure induces a marked fluorescence enhancement that remains reversible between 1 atm and 1.2 GPa due to pressure-driven AIE and exciton recombination (Figure 8h,i) [68]. These findings highlight that the piezochromism of CDs primarily arises from pressure-modulated interparticle interactions and surface-state reorganization, offering a low-toxicity, mechanically sensitive alternative to traditional semiconductor QDs for applications in flexible sensors, anticounterfeiting, and pressure-responsive optoelectronics.

## 4. Applications of Piezochromic Nanomaterials

### 4.1. Pressure-Tunable Light Sources: Lasers and LEDs

Pressure offers a powerful and inherently clean stimulus for modulating light emission in nanoscale luminophores, owing to its ability to precisely tune molecular conformation, intermolecular interactions, electronic coupling, and charge transfer (CT) pathways [70,71,72]. Unlike chemical doping or thermal treatments, pressure manipulation does not introduce chemical impurities and can simultaneously regulate both fluorescence and phosphorescence channels by altering excited-state landscapes. Across quantum dots, metal–organic frameworks, organic emitters, and hybrid crystals, pressure has been shown to narrow or widen bandgaps, activate dark excitons, enhance spin–orbit coupling, and strengthen radiative transitions. This tunability enables continuous or stepwise shifts in emission color, intensity enhancement in initially weakly emissive systems, and the generation of multi-color or even white-light outputs within a single material platform [40,73,74]. Such mechanically programmable emission modes offer distinct advantages for adaptive optical sources, tunable micro-lasers, wavelength-reconfigurable LEDs, and pressure-encoded optical communication components.

**Figure 9:**
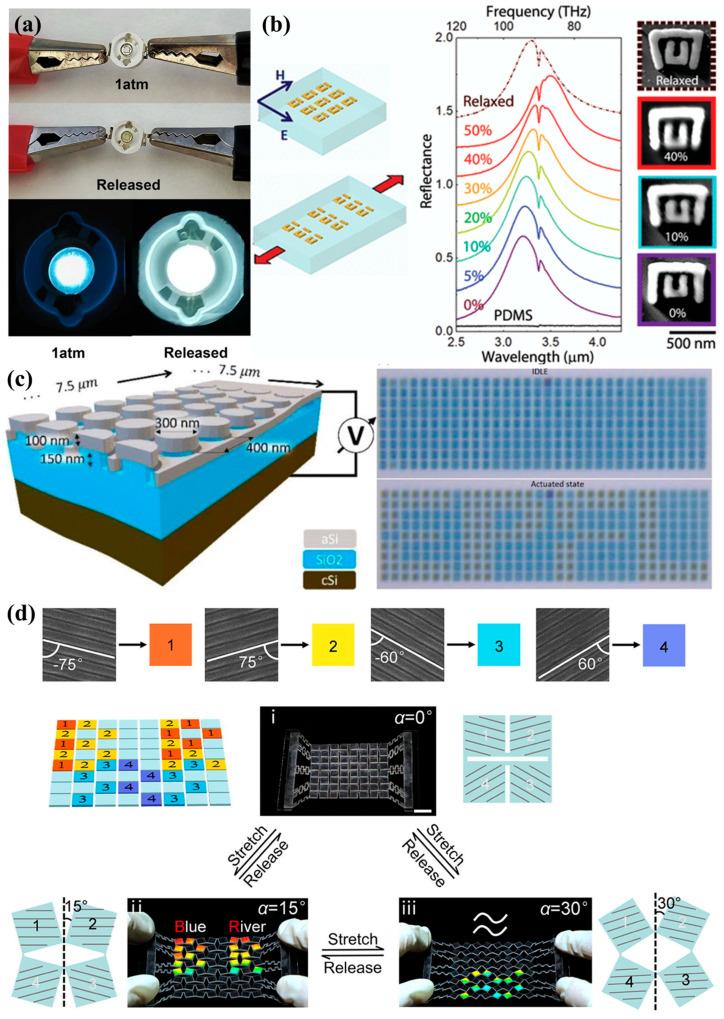
(**a**) Photographs of the operating white pc-LED device. Reproduced with permission [75]. Copyright 2025, Springer Nature. (**b**) Highly Strained Compliant Optical Metamaterials with Large Frequency Tunability. Reproduced with permission [76]. Copyright 2010, American Chemical Society. (**c**) Demonstration of the modulation capability showcasing the EPFL logo when actuated state. Reproduced with permission [77]. Copyright 2023, American Chemical Society. (**d**) Grating patterns with different azimuth angles (illustrated by code patterns) and the process of reading encrypted patterns by stretching. Reproduced with permission [78]. Copyright 2023, Science China Press.

A representative demonstration of pressure-engineered light sources is offered by the recently reported Cd(BDC)(DMF) framework, where multi-level pressure treatments turn an initially faint emitter into a bright blue–green–white light source. Compression stabilizes the linker conformation, enhances hydrogen-bond networks, restricts long-range CT, and increases radiative oscillator strength. Importantly, engineered CT pathways—featuring nearly orthogonal natural transition orbitals—facilitate efficient intersystem crossing, enabling simultaneous fluorescence–phosphorescence regulation and pressure-triggered multicolor emission. Remarkably, the multi-color luminescence remains stable and fully recoverable upon pressure release, a capability rarely observed in stimuli-responsive PL systems. Utilizing this strategy, a bright white phosphor-converted LED (pc-LED) was fabricated by integrating pressure-treated Cd(BDC)(DMF) onto a 365 nm UV chip (Figure 9a). The device exhibited stable CIE coordinates (0.29, 0.34), robust emission across 10–90 mA driving currents, and excellent fatigue stability for over 72 h—demonstrating the feasibility of pressure-engineered emitters for practical optoelectronic applications [75]. Looking forward, pressure-responsive emitters hold potential for miniaturized tunable lasers, flexible color-reconfigurable displays, optical encryption, and smart photonic systems where mechanical cues serve as an additional degree of freedom for dynamic light manipulation.

### 4.2. Reversible Pressure-Tunable Photonic Crystals & Metasurfaces

Mechanical pressure provides an effective and reversible means of tuning the optical response of photonic crystals and metasurfaces by directly modulating their subwavelength structural parameters. In contrast to conventional passive nanophotonic architectures, where resonant properties are permanently fixed by lithographically defined unit-cell geometries—mechanical deformation introduces an additional dynamic degree of freedom that enables in situ control of optical dispersion. Applying hydrostatic or uniaxial pressure can compress inter-element spacing, alter lattice constants, or deform dielectric or plasmonic resonators, leading to predictable blue-shifts or red-shifts in photonic bandgaps and resonance wavelengths [79,80,81]. When implemented on elastomeric or mechanically compliant substrates, these systems can support large, reversible strain, providing continuous tunability across broad spectral windows while maintaining structural integrity. Such strain-engineered metasurfaces serve as a promising platform for reconfigurable nano-optics, adaptive focusing, and tunable spectral filtering.

A compelling demonstration of this concept is seen in mechanically compliant metasurfaces integrated onto elastomeric substrates (Figure 9b). By controllably modifying the spacing between resonant elements through high-strain deformation, tunability as large as Δλ ≈ 400 nm has been achieved—far exceeding the intrinsic resonant linewidth at optical frequencies. This unprecedented tuning enables functionalities such as dynamically switching surface-enhanced infrared absorption (SEIRA), where pressure-driven resonance alignment with the CH stretching mode yields a 180-fold enhancement in reflection intensity. Additionally, mechanical manipulation of coupled resonator components makes it possible to modulate Fano resonances with high precision, offering new opportunities for pressure-driven optical encoding, refractive index sensing, and force-responsive spectroscopic interfaces [76]. Looking forward, pressure-tunable photonic crystals and metasurfaces are expected to play an increasingly important role in nano-optoelectronic systems, where reversible mechanical actuation can be harnessed for miniature spectrometers, pressure-encoded optical memories, and adaptive photonic elements capable of real-time spectral reconfiguration.

### 4.3. Piezo-Phototronic Enhancement and Pressure-Modulated Optical Modulators

Mechanical modulation has become an increasingly attractive strategy for constructing adaptive nano-optoelectronic systems, particularly where electrical or thermal tuning is limited by bandwidth, device complexity, or power consumption. Pressure-responsive nanomaterials and mechanically reconfigurable metasurfaces provide a direct pathway for dynamic control of optical amplitude, phase, and spectral position in ultracompact devices [82]. Their compatibility with flexible substrates and MEMS architectures further supports low-power operation and real-time reconfigurability, offering new opportunities for integrated photonics, portable imaging systems, and next-generation reflective or emissive displays [77].

In one implementation, a mechanically tunable metasurface reflectivity modulator is realized using an amorphous silicon nanopillar array coupled to a suspended silicon membrane with integrated electrostatic actuators (Figure 9c). A membrane displacement of only ~150 nm is sufficient to produce broadband reflectivity modulation across 400–530 nm, yielding a contrast ratio of approximately 1:3. This tunability originates from mechanically shifting the membrane to alter Mie-resonance-enhanced absorption within the nanopillar ensemble, enabling precise amplitude control of reflected light. With its fast, low-power actuation and wide visible-range response, this pressure-modulated metasurface design is well suited for high-frame-rate reflective displays, compact optical modulators, and adaptive photonic components [77]. More broadly, such mechanically tunable systems highlight the promise of strain-mediated optical control as a complementary route to piezo-phototronic enhancements in nanowire-based LEDs, photodetectors, and strain-gated optoelectronic devices.

### 4.4. High-Sensitivity Optical Pressure Sensing, Anti-Counterfeiting & Information Security

The integration of piezochromic nanomaterials into security and sensing architectures enables mechanical stimuli to function as an additional degree of freedom for optical information modulation. In particular, multichannel information encryption—where multiple independent optical states are encoded within a single platform—has emerged as a powerful strategy for enhancing data density and security. Existing multichannel systems based on metasurfaces, photonic crystals, and hybrid optical structures typically operate by modulating the optical degrees of freedom such as polarization, angle, or wavelength, or by mechanically reconfiguring micro- and nanoscale patterns [83,84,85]. Piezochromic materials add a unique dimension to this framework: their color, intensity, and spectral response can be reversibly altered by pressure, enabling pressure-gated readout, orthogonal decryption pathways, and physically unclonable optical signatures. Such capabilities are particularly important for high-security anti-counterfeiting, tamper-evident packaging, mechanical authentication, and covert information storage.

A versatile implementation of this concept draws inspiration from structural color changes in biological systems such as fish scales and bird feathers (Figure 9d). By employing a deformable two-dimensional “fog-inspired” architecture in which the orientation of 1D grating arrays is governed by topological spatial deformation, rapid, repeatable, and programmable color switching is achieved. Unlike conventional mechanochromic or bioinspired systems—where color change typically arises from expansion or contraction of photonic lattices, this strategy modulates structural color by in-plane rotation of grating elements, altering the output angle of reflected light without damaging fine optical structures. The resulting grating sheet supports mechanically driven multichannel encryption, offering high-level information security through spatial–spectral encoding, pressure-triggered decryption, and robust cycling stability. This deformable grating platform exemplifies how mechanically tunable optical architectures can enable next-generation anti-counterfeiting, soft camouflage, and adaptive information display technologies [78].

### 4.5. Piezochromism-Enabled Strategies for Advanced Solar Cell Design

#### 4.5.1. Mechanisms and Multifunctional Potential

The multifunctionality of piezochromic nanomaterials is fundamentally rooted in their unique physical response mechanisms to external mechanical stimuli. This characteristic provides a critical materials-science pathway for developing next-generation “smart” photovoltaics by directly converting mechanical stress and strain into controllable modifications of electronic structures, crystal lattices, or molecular arrangements. Through these pathways, mechanical inputs are translated into precise changes in optical properties, such as absorption edges and emission spectra, alongside altered electrical characteristics like bandgap energy and carrier mobility. Consequently, these materials bestow an unprecedented degree of environmental adaptivity and functional extensibility upon photovoltaic (PV) devices that traditional designs cannot achieve. Specifically, stress-induced bandgap modulation allows photoactive layers to optimize their spectral response based on real-time irradiance, maximizing energy harvesting in diverse climates. Simultaneously, the visible color transitions or fluorescent responses triggered by applied pressure serve as built-in, non-contact optical sensing signals. This enables in situ, real-time monitoring of the mechanical structural integrity of PV modules, providing early warning and localization for microcrack initiation and the accumulation of fatigue damage. Furthermore, integrating stress-triggered signals with self-healing architectures allows systems to proactively repair damage, extending device lifespan in flexible and wearable applications [12,86,87].

From a systemic perspective, these three functional dimensions—self-optimizing performance, real-time structural health monitoring, and active lifetime extension—are not isolated but instead synergistically construct a closed-loop intelligent management system. Performance self-optimization transforms solar cells from passive energy-conversion units into active systems capable of dynamically adjusting their operational states according to the external lighting environment, ensuring globally optimal energy output at the system level. The built-in mechanical health monitoring provides a low-cost, visualized, and circuit-free preventive maintenance solution for photovoltaic systems, especially in large-scale power plants and Building Integrated Photovoltaics (BIPV)—effectively reducing the risks of sudden downtime and the maintenance costs associated with latent mechanical failures. Ultimately, the intrinsic potential for extending device longevity points toward a more resilient and sustainable photovoltaic design philosophy. By leveraging the intelligent response of the material itself to anticipate, mitigate, or offset damage induced by stress accumulation, this approach establishes a solid foundation for high-reliability, long-life photovoltaic solutions tailored for complex and dynamic operational environments, including wearable electronics, curved vehicle roofs, and flexible portable devices.

#### 4.5.2. Experimental Validation and Case Studies

**Figure 10:**
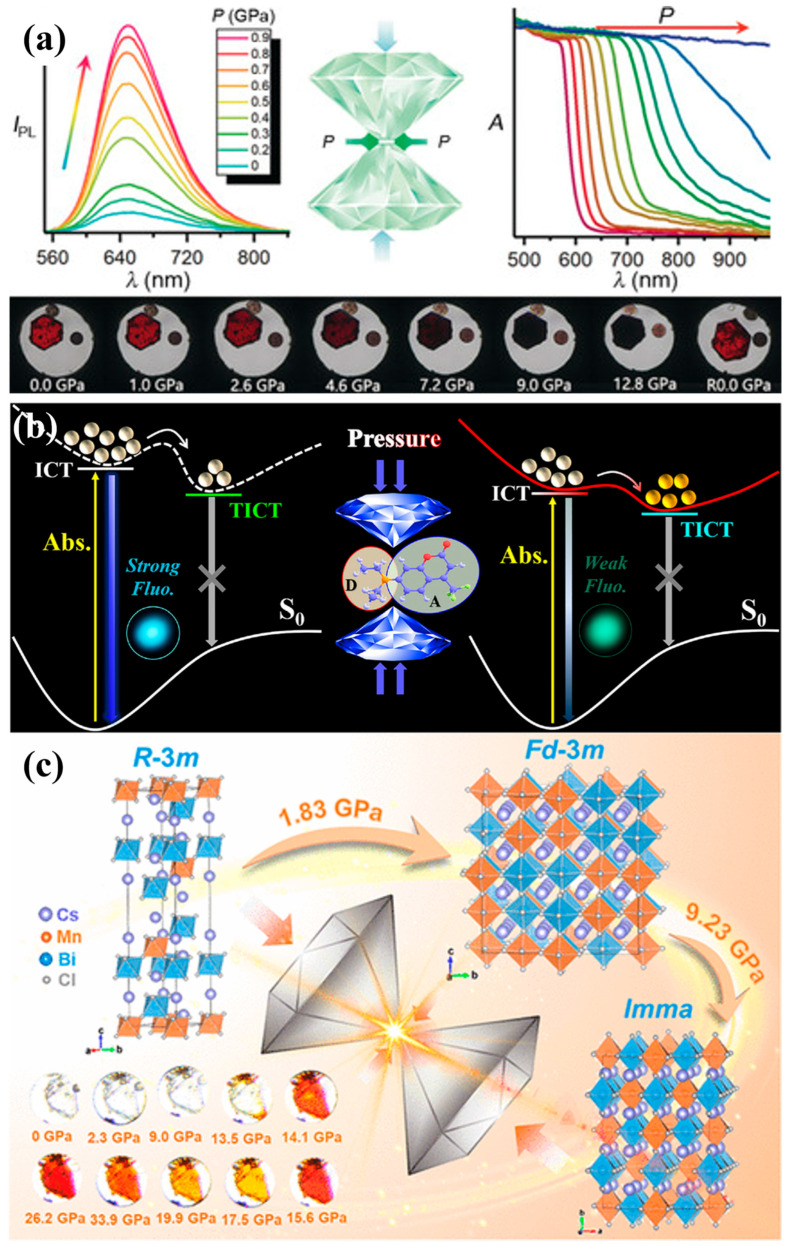
(**a**) Pressure-Induced Emission Enhancement, Bandgap Narrowing, and Metallization of Halide Perovskite Cs_3_Bi_2_I_9_. Reproduced with permission [7]. Copyright 2018, Springer Nature. (**b**) Schematic of the ultrafast dynamics of C35 at atmospheric pressure and under compression. Reproduced with permission [8]. Copyright 2025, Wiley-VCH Verlag. (**c**) Pressure-Induced Piezochromism and Structure Transitions in Lead-Free Layered Cs_4_MnBi_2_Cl_12_ Quadruple Perovskite. Reproduced with permission [9]. Copyright 2021, American Chemical Society.

Recent materials-level investigative studies illustrate how pressure-regulated optical transitions directly benefit photovoltaic functionality. For example, zero-dimensional lead-free perovskite Cs_3_Bi_2_I_9_ (Figure 10a) shows reversible bandgap narrowing under modest lattice compression, driven by Bi–I bond contraction and reduced Bi–I–Bi angles that enhance orbital overlap. Such pressure-induced narrowing enables access to bandgaps approaching—or even surpassing—the Shockley–Queisser limit while simultaneously increasing emission intensity through strengthened exciton binding. At higher pressures, Cs_3_Bi_2_I_9_ undergoes a semiconductor-to-metal transition, revealing that piezochromic structural distortions can drastically reshape carrier transport pathways, offering theoretical guidance for strain-engineered lead-free perovskite photovoltaics [7].

Mechanical modulation has also been explored in the dye-sensitized solar cell (DSSC) field. Pressurizing surfactant–dye assemblies dramatically reduce the critical micelle concentration of CTAB—by more than 100-fold at 0.80 GPa—while stabilizing dye microenvironments (Figure 10b). Pressure-altered charge transfer dynamics enhance dye stability and light-harvesting efficiency, bridging piezochromic behavior with photovoltaic performance [8].

High-pressure engineering has further been applied to lead-free quadruple halide perovskites such as Cs_4_MnBi_2_Cl_12_, which exhibit two-step bandgap narrowing and a sharp piezochromic transition associated with pressure-induced structural phase changes (Figure 10c). These mechanically triggered electronic transitions highlight the theoretical potential of piezochromic pathways to tune wide-bandgap absorbers into the optimal regime for tandem architectures or high-stability inorganic photovoltaics [9].

#### 4.5.3. Application Prospects and System Integration

Integrating piezochromic nanomaterials into photovoltaic architectures enables a transition from static converters to adaptive “smart” systems with enhanced environmental resilience. To evaluate the practical viability of these systems, it is essential to bridge the gap between high-pressure laboratory studies and operational stressors. While diamond-anvil-cell research often explores the GPa regime to probe fundamental phase transitions, realistic photovoltaic modules encounter significantly lower mechanical loads, such as wind pressure, encapsulation-induced shrinkage, and thermal expansion mismatches, which typically operate at the kPa or MPa scale. Despite this pressure discrepancy, certain piezochromic mechanisms remain active at these operational levels. Soft-lattice systems possess favorable mechanical compliance, allowing MPa-level stresses to modulate exciton-phonon coupling and STE dynamics [17,88]. Furthermore, the concentration of strain at heterogeneous interfaces between absorbers and charge transport layers can generate local effective pressures that activate piezochromic pathways far below bulk thresholds [89]. By leveraging these inherent relationships, next-generation solar cells can achieve strain-programmable functionality, where performance parameters like open-circuit voltage are dynamically tuned via internal or external mechanical loads. This capacity for autonomous adaptation allows absorbers to mitigate the impacts of heterogeneous stresses—such as those originating from thermal cycling—while facilitating the stabilization of photoactive phases.

Beyond performance optimization, the deployment of piezochromic nanomaterials fosters a “closed-loop” intelligent management paradigm that integrates high-efficiency energy generation with proactive diagnostic and aesthetic capabilities. Specifically, the visible color transitions or fluorescent spectral shifts triggered by mechanical deformation serve as built-in, non-contact sensors for in situ structural health monitoring, enabling the early detection of microcracks or interfacial delamination to significantly reduce maintenance costs for large-scale solar farms and building-integrated photovoltaics. Furthermore, combining piezochromic layers with piezoelectric and IoT sensors enables hybrid architectures that capture both solar and mechanical energy while providing real-time visual feedback.

## 5. Challenges and Future Directions of Piezochromic Nanomaterials in Solar Cell Applications

**Figure 11:**
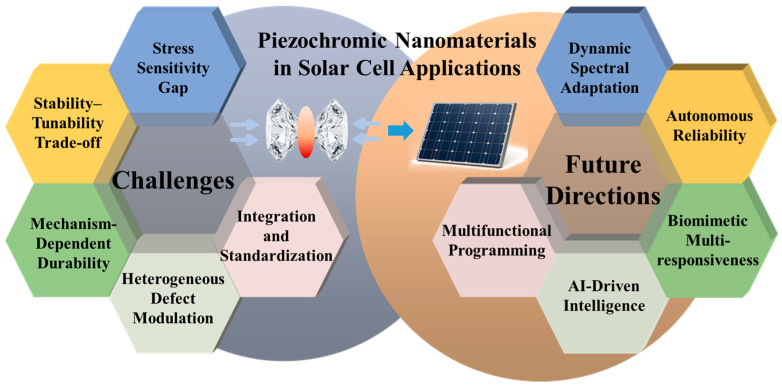
Challenges and Future Directions of Piezochromic Nanomaterials in Solar Cell Applications.

As shown in Figure 11, piezochromic nanomaterials offer significant potential for strain-programmable bandgap tuning and adaptive photovoltaics. However, translating laboratory high-pressure phenomena into operational solar cells is challenging. Practical PV devices encounter much lower and more heterogeneous stresses than those used in laboratory settings. Furthermore, absorbers must maintain structural stability, defect passivation, and long-term durability under continuous thermal and mechanical cycling. The following sections detail these challenges and outline prospective pathways toward fully exploiting piezochromic mechanisms in next-generation photovoltaic technologies.

### 5.1. Challenges

#### 5.1.1. Decoupling of Laboratory Phenomena from Realistic Device Stress Environments

A primary challenge lies in the sensitivity mismatch between laboratory discovery and industrial utility. Many profound piezochromic effects—such as bandgap narrowing, exciton stabilization, and defect reorganization—are typically observed under laboratory compression conditions (often in the GPa range) that far exceed the intrinsic stresses experienced in operating PV devices. In practice, solar cells undergo only modest mechanical perturbations originating from thermal expansion mismatch, grain-boundary stress, or encapsulation-induced contraction. Consequently, the strongest piezochromic mechanisms often do not directly translate to realistic device environments where only mild strain is available. Bridging this gap requires engineering materials with low-pressure responsiveness, such as soft-lattice perovskites or the use of stress concentrators to locally magnify small deformations.

#### 5.1.2. Intrinsic Trade-Off Between Optoelectronic Tunability and Structural Stability

This challenge is particularly acute in halide perovskites, where the soft ionic lattice that enables excellent piezochromic tunability also results in mechanical fragility and susceptibility to ion migration. While compressive strain can beneficially narrow the bandgap, it may simultaneously promote halide vacancy migration or trigger irreversible lattice collapse in lead-free alternatives. These competing effects create a fundamental stability–strain trade-off. Future efforts must prioritize strain-tolerant and fatigue-resistant absorber materials, such as high-entropy perovskites or compositions incorporating flexible organic moieties that cushion lattice deformation without compromising phase stability. For instance, drawing inspiration from flexible electronics, Kirigami-patterned architectures used in piezoelectric sensors have demonstrated the ability to greatly expand the viable strain range while maintaining stable functional performance under mechanical deformation [90,91]. Similarly, customized Kirigami electrodes have been developed for flexible and deformable lithium-ion batteries, enabling high levels of stretchability and structural resilience during extreme mechanical deformation [92].

#### 5.1.3. Interdependence of Reversibility, Fatigue Resistance, and Microscopic Mechanisms

The durability of piezochromic behavior is fundamentally defined by the underlying physical mechanisms. Materials governed by weak intermolecular interactions, such as van der Waals forces, often display highly reversible responses because structural perturbations are elastic and readily recoverable. In contrast, systems involving significant structural reorganization—such as covalent framework distortion or pressure-induced phase transitions—are more prone to hysteresis and kinetic barriers. For PV applications, ensuring that optical properties retrace their pathway during decompression is critical to avoid performance decay over 10^5^–10^6^ cycles of thermal and mechanical loading.

#### 5.1.4. Complex Modulation of Defect Energetics and Carrier Dynamics

While mechanical stress can reorganize defect states and suppress non-radiative recombination, these effects are limited by the heterogeneous strain fields found in polycrystalline thin films. Dislocations, grain boundaries, and vacancies introduce a spatially variable defect landscape, leading to inconsistent electronic responses when mechanical perturbations are applied. To master strain-mediated defect control, researchers must utilize operando techniques—such as PL/Raman mapping and time-resolved microwave conductivity—to visualize the dynamic interplay between strain and carrier transport. This is essential for developing absorbers that can self-optimize during operation by leveraging internal stress to maintain high carrier lifetimes.

#### 5.1.5. Technical Hurdles in Device Integration and Lack of Standardization

Integrating piezochromic materials into practical architectures remains a major engineering challenge due to the mechanical mismatch among transport layers, encapsulation, and substrates. Such mismatch complicates the transfer of beneficial strain into the absorber and can lead to long-term fatigue or fracture. Furthermore, the field lacks universal metrics to evaluate piezochromic performance across different material classes under standardized conditions. Future progress relies on adopting mechanically adaptive architectures, such as strain-amplifying substrates and stress-buffering interlayers, while establishing quantitative models to maximize efficiency gains through strain-optoelectronic coupling.

### 5.2. Future Directions

#### 5.2.1. Dynamic Spectrally Adaptive Systems for Intelligent Photovoltaics

A key frontier is the development of “smart” solar cells that autonomously adapt to fluctuating environmental irradiance. By integrating piezochromic materials into the active layer or encapsulation matrix, devices can harness environmental stressors—such as cyclic thermal expansion and wind pressure—to adaptively modulate their absorption spectra. For instance, the system could broaden its absorption range under low-light conditions to maximize photocurrent, while shifting the absorption edge under intense solar radiation to mitigate thermalization losses and optimize open-circuit voltage. Future research must prioritize the discovery of material systems characterized by low-trigger stress thresholds, rapid response kinetics, and robust cycling stability to ensure effective all-weather thermal management and efficiency optimization.

For instance, a system could broaden its absorption range under low-light conditions to maximize photocurrent, while shifting the absorption edge under intense radiation to mitigate thermalization losses and optimize open-circuit voltage. Future research must prioritize material systems with low-trigger stress thresholds, rapid response kinetics, and robust cycling stability to ensure effective all-weather efficiency optimization.

#### 5.2.2. High-Reliability Photovoltaic Devices with Self-Sensing and Self-Healing Capabilities

The integration of the “stress visualization” functionality inherent in piezochromic materials with advanced encapsulation and self-repair technologies offers a pathway toward highly reliable photovoltaic modules. In this framework, piezochromic units serve as embedded mechanical sensors that provide real-time, visual reporting of early-stage structural degradation, such as microcracks or interfacial delamination, through discernible changes in color or photoluminescence signatures. This optical feedback can be further coupled with self-healing mechanisms—such as the stress-triggered release of repair agents from microcapsules—to achieve preventive maintenance. In flexible or wearable photovoltaics, such “smart” layers can act as fatigue-warning indicators, significantly extending device lifespan and reducing long-term operational costs.

#### 5.2.3. Structural Innovation Through Stress-Programming and Multifunctional Integration

Future device architectures should transcend the single function of power generation by exploring multifunctional integration enabled by active stress-programming. Hybrid piezoelectric–piezochromic devices could simultaneously harvest solar and mechanical energy, with the piezochromic response serving as an intuitive indicator of energy conversion states. For BIPV, stress-modulated intelligent facades enable dynamic aesthetic effects and functional visual camouflage. By mechanically regulating reflectivity and color, these panels provide anti-glare properties and enhance social acceptance without compromising photovoltaic efficiency.

#### 5.2.4. AI-Driven Material Discovery and Intelligent Device Operation

The synergy between artificial intelligence (AI) and big data will accelerate the transition of piezochromic photovoltaics from laboratory concepts to industrial reality. Through machine learning algorithms and high-throughput computational screening, researchers can inverse-design novel materials with idealized “stress–optical–electronic” coupling characteristics. At the system level, photovoltaic panels integrated with piezochromic sensors can function as IoT nodes, uploading spatially resolved mechanical health data to the cloud. AI-based analysis of these real-time datasets enables predictive failure modeling and digitization, intelligent operation and maintenance for large-scale solar power plants.

#### 5.2.5. Bio-Inspired and Multi-Stimuli Responsive Next-Generation Energy Materials

Long-term exploration will focus on biomimetic designs inspired by organisms like the chameleon, aiming to create energy materials that respond to a complex array of environmental stimuli. The goal is to develop next-generation smart materials capable of simultaneously sensing and responding to stress, light intensity, temperature, and moisture. Such systems would enable synergistic carrier behavior regulation—for example, utilizing thermal stress from temperature fluctuations alongside light intensity variations to co-optimize exciton dynamics and charge transport. These multi-stimuli responsive systems will provide the foundation for highly adaptive, environment-interactive energy frameworks that go beyond traditional photovoltaic limitations.

## 6. Conclusions

Piezochromic nanomaterials represent a rapidly expanding class of mechanically responsive optical systems whose strain-mediated modulation of band structure, excitonic dynamics, and molecular packing enables tunable and programmable light–matter interactions. Across perovskites, metal halides, organic luminogens, MOFs, and hybrid nanostructures, pressure has been shown to activate reversible color switching, enhance radiative pathways, reorganize defect energetics, and trigger lattice-encoded optical transitions. These advances establish piezochromism as a versatile design principle for adaptive nano-optoelectronic platforms, offering new mechanisms for dynamic emission control, reconfigurable photonics, and mechanically gated optical functionalities.

Beyond fundamental photophysical insights, current research is increasingly translating these mechanisms toward intelligent photovoltaic functions, such as stress-mediated spectral self-adaptation, built-in structural health monitoring, and potential self-healing capabilities. Realizing these promises hinges on overcoming critical translation barriers, including efficient strain transfer in real device stacks, the intrinsic stability–tunability trade-off, and the integration of responsive materials within mechanically mismatched architectures. Addressing these challenges demands a concerted focus on novel material design for low-threshold response, advanced strain engineering at the device level, and the development of intelligent, multifunctional systems for next-generation adaptive solar energy technologies.

## Figures and Tables

**Table 1:** Summary of Piezochromic Mechanisms and Characteristics in Different Material Classes.

Material Class	Perovskites	Metal Halides	Organic Lumingens	Metal–Organic Frameworks
Mechanism(s)	Octahedral distortion; bandgap modulation; exciton–phonon cou-pling	Structural compression in soft ionic lattice; self-trapped exciton mod-ulation	Molecular conformation change; in-termolecular interactions	Framework deformation; host–guest interaction modulation
Pressure Range	1–10 GPa	1–20 GPa	1–10 GPa	0.0001–5 GPa
Quantitative Optical Response	Bandgap narrowing (Mn-doped (PEA)_2_PbBr_4_ NCs exhibit bandgap narrowing from 3.0 to below 2.5 eV under 0–9.2 GPa)	Continuous blueshifts (Cs_3_Sb_2_Br_9_ QDs exhibit a continuous PL blue-shift and rapid intensity enhancement under pressure, peaking at 4.8 GPa)	Large PL shifts (The absorption edge of the TPE-based blue emitter redshifts from 423 to 518 nm under 10.1 GPa)	Typical shifts of 10–80 nm; significant blue-to-red/yellow transitions in specific systems.
Material Morphology	Thin films; nanocrystals	Nanocrystals; quantum dots	Organic crystals; AIE co-crystals	Nanopowders, Host-guest crystals
Reversibility & Cycle Stability	Reversible at moderate pressure; hysteresis increases with phase transitions	Highly reversible due to soft bonding; low hysteresis in 0D systems	Reversible for weak interactions	Often reversible at low pressures; dependent on framework rigidity
Representative Examples	2D perovskites: (PEA)_2_PbBr_4_, (BTa)_2_PbI_4_	Cs_3_Sb_2_Br_9_ QDs; 0D metal halides (Cs_4_PbBr_6_); Mn-doped CsPbBr_3_ NCs	TPE–AN co-crystal; perylene-TCNB co-crystals	Co_2_(Bdc)_2_Dabco·4DMF·H_2_O; Tb(BTC)(H_2_O)_6_
References	[37,38]	[39,40,41]	[17,22,35]	[23,42,43]

## Data Availability

No new data were created or analyzed in this study.
